# A plasmon-driven selective surface catalytic reaction revealed by surface-enhanced Raman scattering in an electrochemical environment

**DOI:** 10.1038/srep11920

**Published:** 2015-07-06

**Authors:** Lin Cui, Peijie Wang, Yurui Fang, Yuanzuo Li, Mengtao Sun

**Affiliations:** 1 Beijing Key Laboratory for Nano-Photonics and Nano-Structure, Center for Condensed Matter Physics, Department of Physics, Capital Normal University, Beijing 100048, People’s Republic of China; 2 Beijing National Laboratory for Condensed Matter Physics, Institute of Physics, Chinese Academy of Sciences, P. O. Box 603-146, Beijing, 100190, People’s Republic of China; 3 Division of Bionanophotonics, Department of Applied Physics, Chalmers University of Technology, Gothenburg SE-412 96, Sweden; 4 College of Science, Northeast Forestry University, Harbin 150040, China

## Abstract

Plasmonic catalytic reactions of molecules with single amine or nitro groups have been investigated in recent years. However, plasmonic catalysis of molecules with multiple amine and/or nitro groups is still unknown. In this paper, plasmon-driven catalytic reactions of 4,4'-dinitroazobenzene (DNAB), 4,4'-diaminoazobenzene (DAAB) and 4-nitro-4'-aminoazobenzene (NAAB) are investigated using electrochemical surface-enhanced Raman scattering (SERS) spectroscopy. The results reveal that a plasmon-driven reduction reaction occurred for DNAB and NAAB in which the NO_2_ group was reduced to NH_2_, while the plasmon-driven oxidation reaction of NH_2_ did not occur. This result demonstrates that plasmon-driven reduction reactions are much easier than plasmon-driven oxidization reactions in electrochemical environments. The molecular resonance may also play an important role in plasmon-driven catalytic reactions. These findings provide us with a deeper understanding of plasmon-driven catalytic reactions.

Plasmonic chemistry^1^, which is based on catalytic reactions driven by surface plasmons, is an important applied field in the study of surface plasmons^2^,^3^,^4^,^5^,^6^. It has been attracting increasing attention since the first reports that p-aminothiophenol (PATP) can be catalyzed to DMAB via surface plasmon resonance in 2010^7^,^8^. Later, it was reported that 4NBT can also be catalyzed to DMAB by surface plasmon resonance^9^,^10^, with hot electrons generated from plasmon decay taking part in this reduction reaction^9^. Such plasmon-driven catalyzed reactions of 4NBT and PAPT into DMAB were further confirmed in tip-enhanced Raman spectroscopy experiments in high vacuum and ambient environments, respectively^2^,^10^,^11^,^12^. The mechanism of plasmon-driven catalytic reactions is mainly attributed to the density of state (DOS) of hot electrons generated from surface plasmon decay. Hot electrons with high kinetic energies play a key role in plasmonic chemistry^10^. This phenomenon is of significant interest in plasmon-driven catalysis, as discussed in several recent excellent reviews^13^,^14^,^15^.

However, in practice, chemical reactions mostly occur in aqueous environments, while the above experimental works were predominantly performed in ambient or vacuum environments^1^,^2^,^3^,^4^,^5^,^6^,^7^,^8^,^9^,^10^,^11^,^12^,^16^. Several experiments have been performed in aqueous environments that show similar catalytic reactions^17^,^18^, and the electrochemical mechanism can work synergistically with plasmon catalysis. The target molecules were NH_2_ or NO_2_ groups in the previous experiments in aqueous environments. If molecules with both NH_2_ and NO_2_ groups are in an aqueous environment, what type of plasmon-driven chemical reaction will occur (i.e., reduction or oxidation)? If the target molecules have two NH_2_ or NO_2_ groups, can the target molecules form dimers via one NH_2_ (or NO_2_) or polymers via two or more NH_2_ (or NO_2_) groups? These questions are very interesting and need to be answered. 4,4'-Diaminoazobenzene (DAAB), 4,4'-dinitroazobenzene (DNAB) and 4-nitro-4'-aminoazobenzene (NAAB) are three good candidates for investigating the above issues, as they possess combinations of these two groups (Fig. 1). DNAB has two NO_2_ side groups, which can be used to study the plasmon-driven reduction reaction; DAAB has two NH_2_ side groups, which can be used to study the plasmon-driven oxidation reaction; and NAAB has both an NH_2_ and an NO_2_ group, so it can be used to study the priority of the plasmon-driven oxidization and reduction reactions.

In this paper, the electrochemical surface-enhanced Raman scattering (SERS) spectra of DNAB, DAAB and NAAB were experimentally studied. We attempt to answer the above questions experimentally and provide an interpretation of the findings based on theory. Plasmon-driven reduction of the NO_2_ groups occurred, i.e., NO_2_ was reduced to NH_2_, while plasmon-driven oxidation of NH_2_ did not occur. Our study provides experimental evidence that the plasmon-driven reduction reaction occurs more easily than the plasmon-driven oxidation reaction in an aqueous environment.

## Results

To have a clear picture of the plasmon catalytic reaction, we must first identify the characteristic Raman peaks of these three molecules (DAAB, DNNB and NAAB). The experimental and calculated Raman spectra of DAAB, DNNB and NAAB powder are shown in Fig. 2. As shown in Fig. 2(a,b), the Raman peak of DAAB at 1398 cm^−1^ is the strongest, representing the –N = N– stretching mode of DAAB. The two Raman peaks (one weak and one strong) at approximately 1152 cm^−1^ are attributed to the asymmetric and symmetric vibrations of H on the two benzyls. The two weak Raman peaks at approximately 1600 cm^−1^ are the asymmetric and symmetric scissor vibrations for the H of the two NH_2_ groups. Figure 2(c,d) reveal that the Raman peak of DNAB at 1350 cm^−1^ is the two symmetric −NO_2_ stretching mode of DNAB. The strongest Raman peak at 1334 cm^−1^ in Fig. 2(e,f) is the −NO_2_ stretching mode of NAAB.

Figure 3 shows the potential-dependent electrochemical SERS spectra of DAAB. The Raman profiles are stable, and they do not change with the variation in the external electric voltage. The weak Raman peak to the left of 1152 cm^−1^ (the asymmetric vibrations of H on the two benzyls) gradually increased as the potential increased, and its Raman strength is comparable to another Raman peak at approximately 1152 cm^−1^. The two weak Raman peaks from the asymmetric and symmetric scissor vibrations of the H atoms in the two NH_2_ groups at approximately 1600 cm^−1^ decreased owing to the increase in the width of the Raman peak. The results show that no reduction or oxidation reaction occurs during the measurement. For further confirmation of this result, please see Supporting Information Fig. S1.

**Figure 1:**
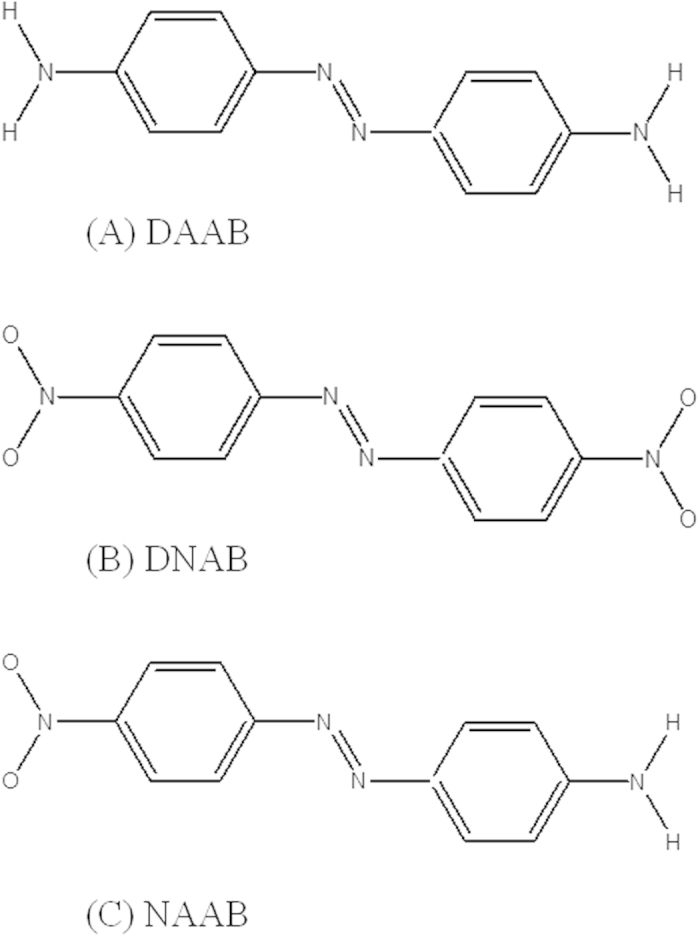
Molecular structures of DAAB, DNAB and NAAB.

**Figure 2:**
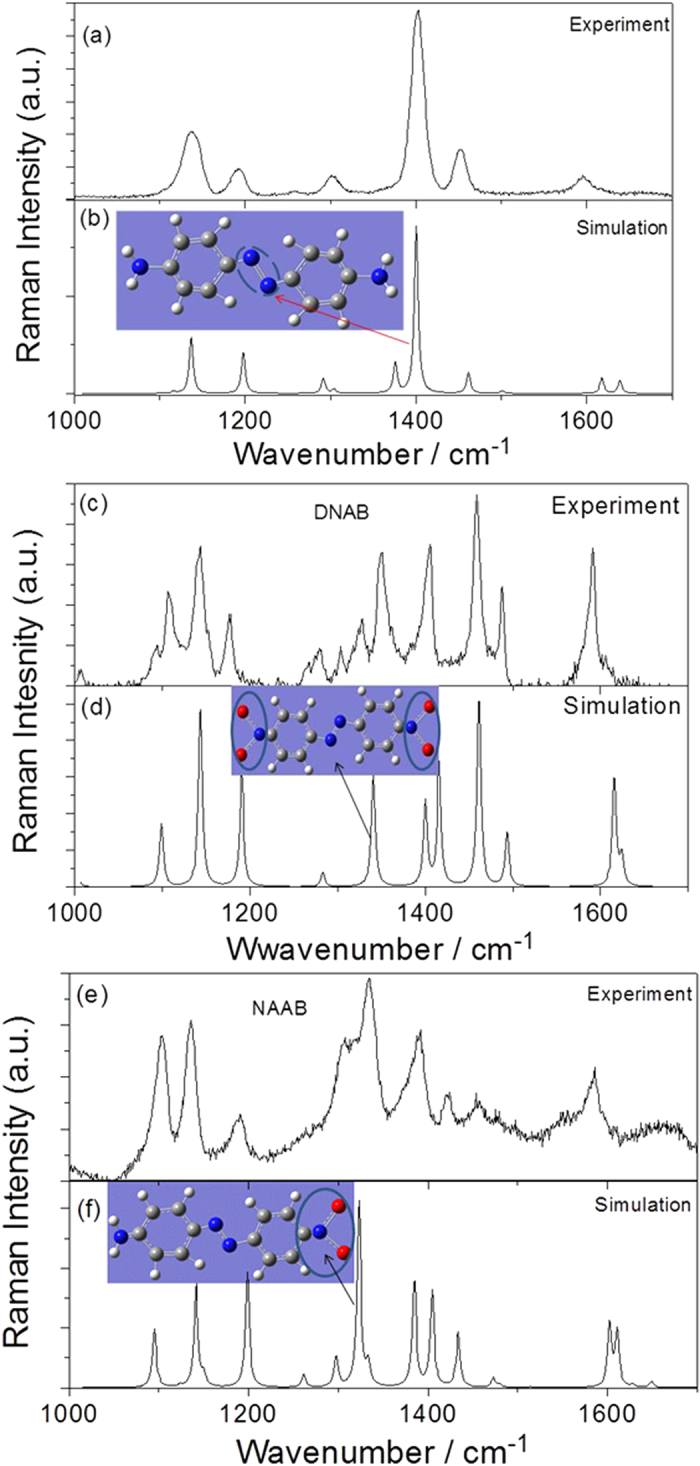
Experimental and theoretical Raman spectra. (**a**) and (**b**) experimental and theoretical DAAB Raman spectra; (**c**) and (**d**) experimental and theoretical DNAB Raman spectra; and (**e**) and (**f**) experimental and theoretical NAAB Raman spectra.

**Figure 3:**
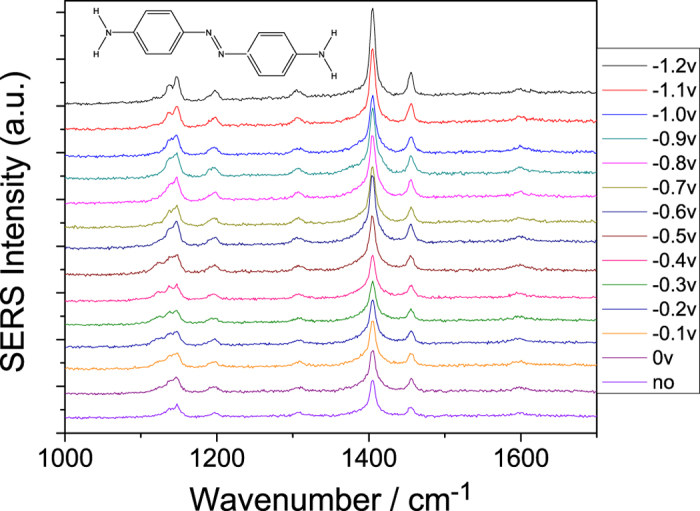
Electrochemical SERS spectra of DAAB at different potentials, excited by a 532 nm laser.

**Figure 4:**
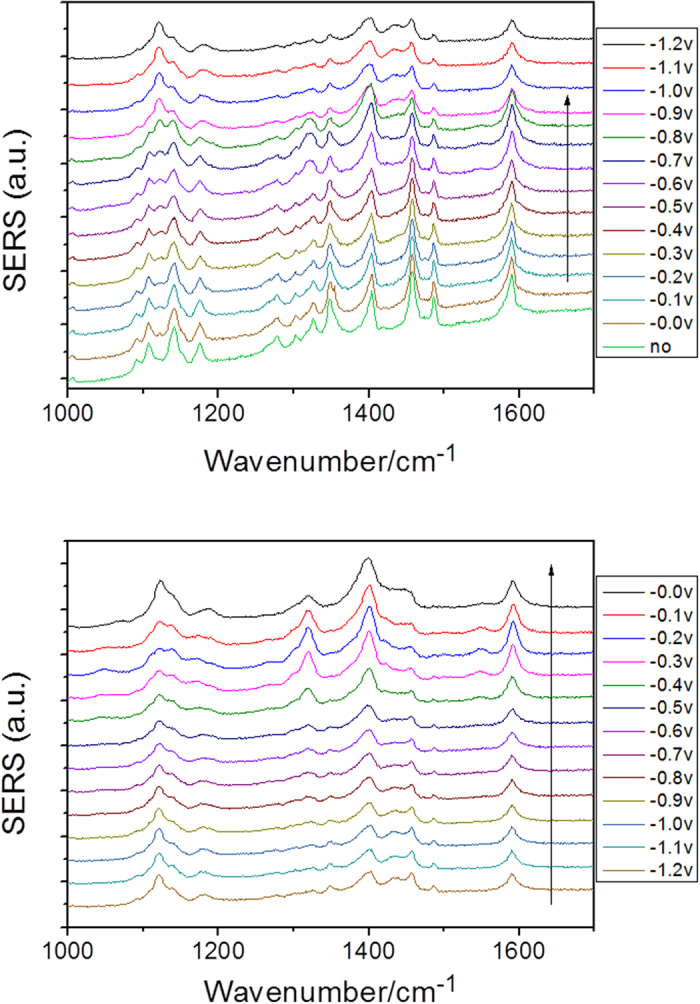
Electrochemical SERS spectra of DNAB at different potentials, excited by a 532 nm laser.

**Figure 5:**
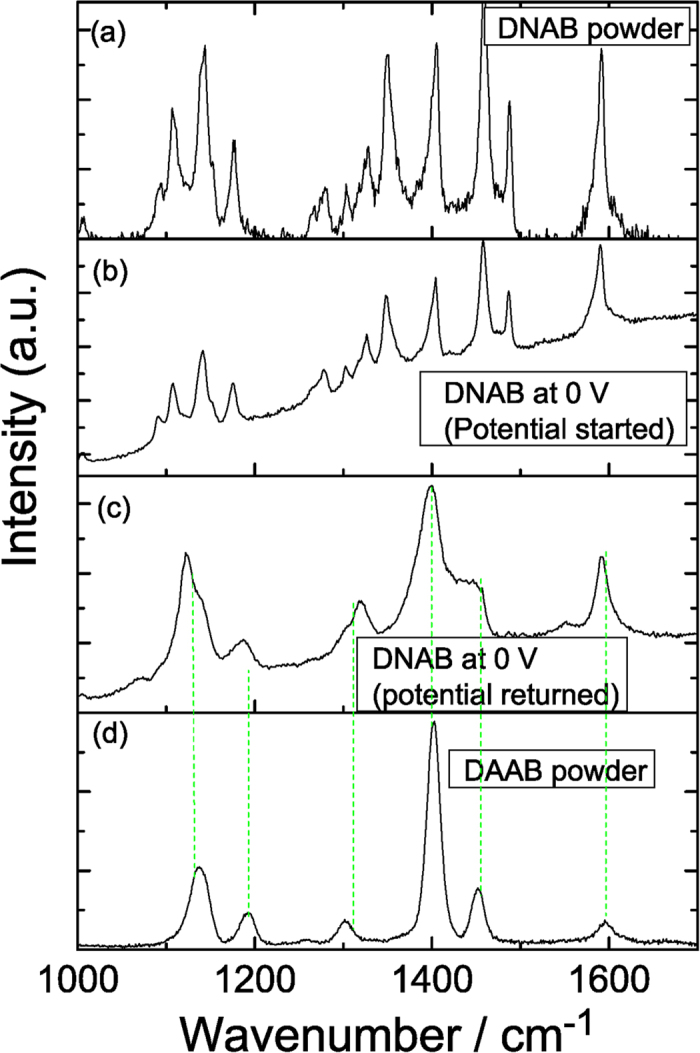
Comparison of the experimental and theoretical Raman spectra of DNAB.

**Figure 6:**
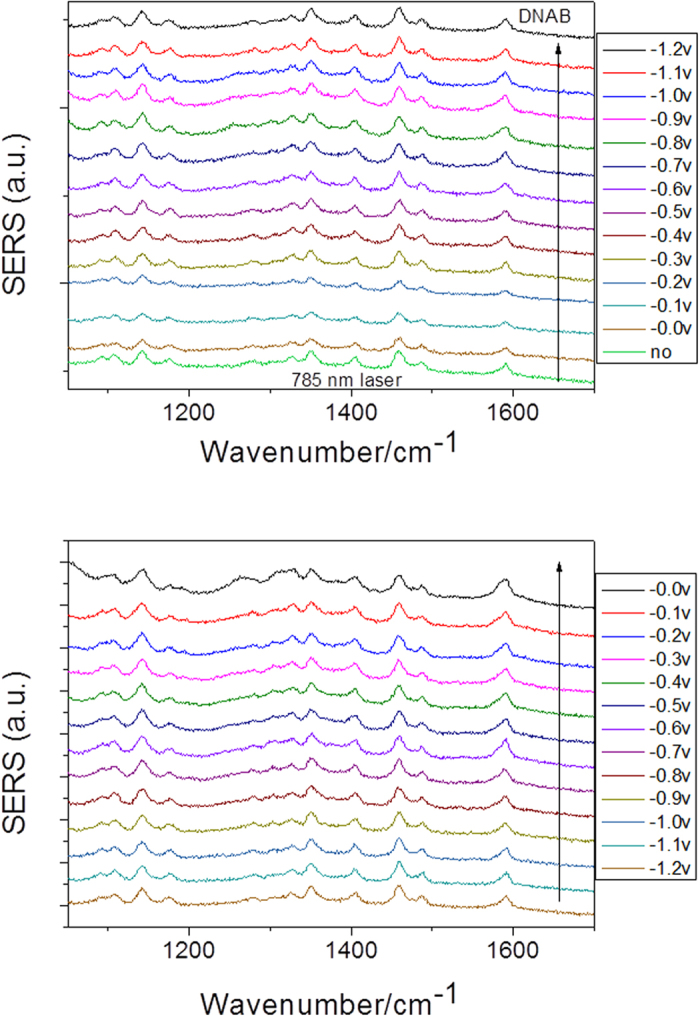
Electrochemical SERS spectra of DNAB at different potentials excited at 785 nm.

Figure 4(a,b) are the potential-dependent electrochemical SERS spectra of DNAB measured with a 532 nm laser. From Fig. 4(a), we can see that the Raman peak at 1350 cm^−1^ gradually decreased as the potential increased, indicating that the two NO_2_ groups of DNAB were reduced by the surface plasmon catalytic reaction. The results indicate that the plasmon-driven chemical reaction occurred during the measurement with the variation of the external electric voltage. The SERS spectra as the external electric voltage returned from −1.2 V to 0 V are shown in Fig. 4(b). The spectra had very similar features during the return of the potential. A comparison is made between the reacted DNAB and the original DAAB (Fig. 5). From the figure, we can see that, at 0 V, there was no chemical reaction, but once the potential increased to −1.2 V (and when it returned to 0 V), the SERS profiles were significantly different. The reacted Raman spectrum of DNAB is the same as the Raman spectrum of DAAB, which indicates that plasmon-driven chemical reactions occurred because of the variation in the external electric voltages, and DNAB was catalyzed to form DAAB by surface plasmon resonance. To reveal the contribution of the surface plasmons to the catalytic reaction, potential-dependent SERS spectra were also measured using a 785 nm laser (see Fig. 6). No reaction occurred during excitation by the 785 nm laser. This means that surface plasmons play a significant role in the reaction, indicating that it is a plasmon-driven chemical reaction because 785 nm is far from the surface plasmon resonance, as shown in the spectrum of the surface plasmon resonance in Fig. 1(d) in reference^18^.

**Figure 7:**
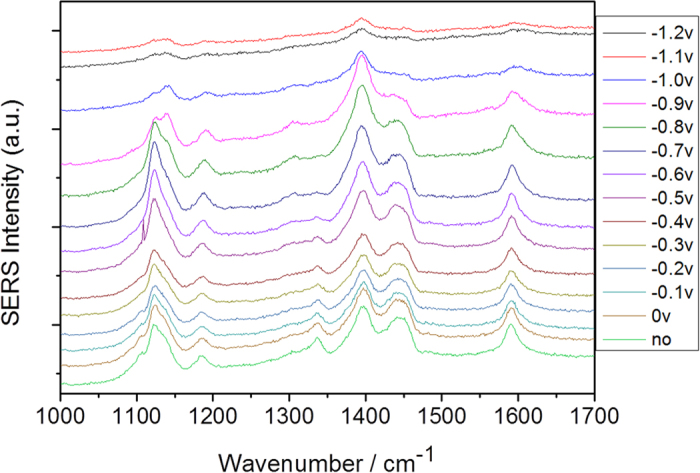
Electrochemical SERS spectra of NAAB at different potentials, excited by a 532 nm laser.

**Figure 8:**
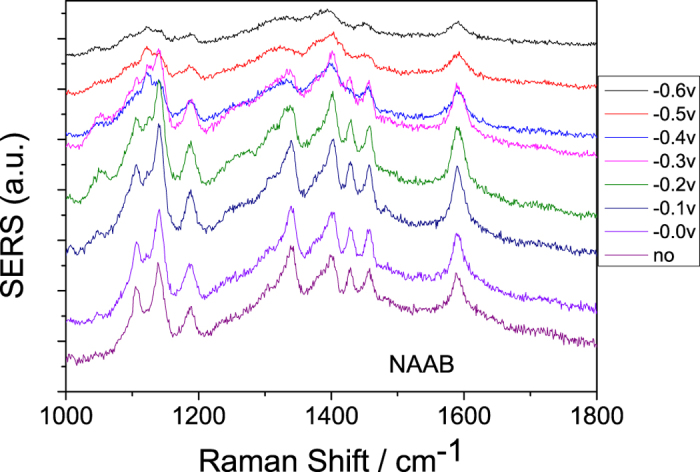
Electrochemical SERS spectra of NAAB at different potentials, excited by a 785 nm laser.

**Figure 9:**
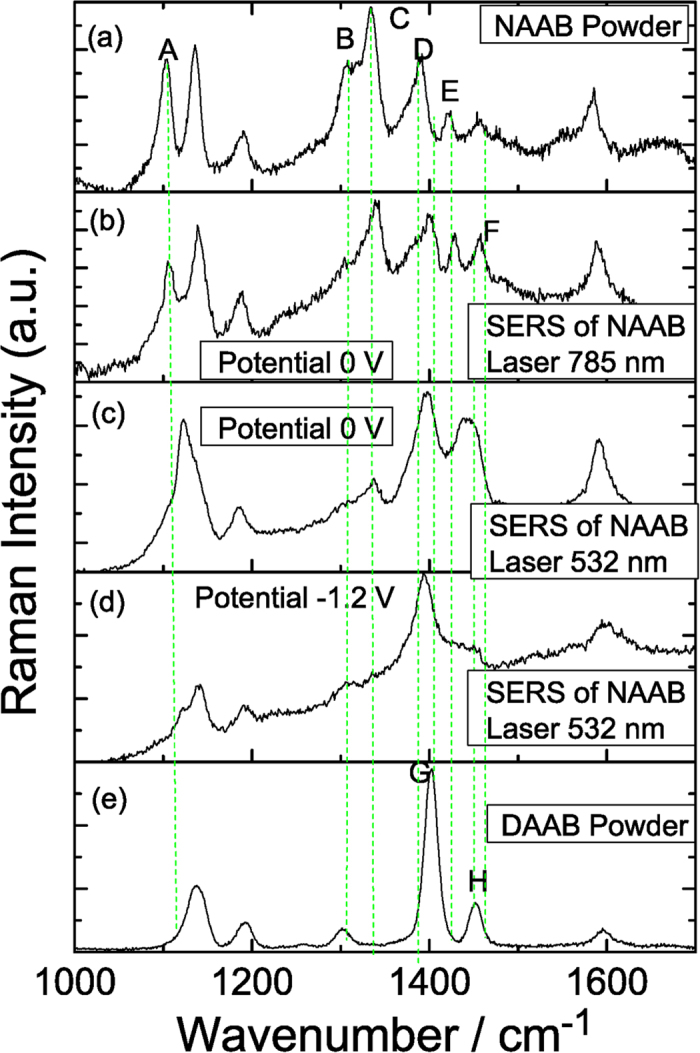
Comparison of the experimental and theoretical Raman spectra of NAAB.

The above experiments show the plasmon-driven reaction conditions of DAAB (or DNAB), which has two NH_2_ (or NO_2_) groups. However, for NAAB, which has both NH_2_ and NO_2_ groups, what will happen? The potential-dependent electrochemical SERS spectra of NAAB were measured using a 532 nm laser (see Fig. 7) and a 785 nm laser (see Fig. 8). As shown in Fig. 7, the profiles of the potential dependent SERS spectra are significantly different at the different electric voltages when excited by the 532 nm laser, which reveals that a chemical reaction occurred. Figure 9(a) is the Raman spectrum of NAAB powder, and Fig. 9(b) is the SERS spectrum of NAAB at 0 V excited by a 785 nm laser. Upon comparing Fig. 9(a,b), we can see that their profiles are almost the same. Figure 9(c,d) are the SERS spectra of NAAB measured at 0 V and −1.2 V, excited by the 532 nm laser. By comparing Fig. 9(b,c), we can see that even at 0 V, upon excitation by the 532 nm laser, the plasmon-driven chemical reaction occurred due to the strong SPR peak at approximately 532 nm, as the Raman peak at 1350 cm^−1^ (−NO_2_ vibration) significantly decreased, although it did not completely disappear. By comparing Fig. 9(d,e), we can see that the SERS spectrum at −1.2 V is almost the same as that of the DAAB powder, indicating that NAAB was catalyzed to DAAB in this system by surface plasmon resonance.

## Discussion

In the sequences of the potential-dependent electrochemical SERS spectra, we can see that, for DNAB, the reaction occurs between −0.8 and −0.9 V, which means that the surface plasmon energy associated with the applied potential can allow the electrons to overcome the barrier and drive the reaction upon excitation with a 532 nm laser. For NAAB, the reaction can occur at 0 V, which means that the reaction has a much lower barrier; upon excitation with the 532 nm laser, the energy of the surface plasmon is enough for the catalytic reaction. All of the experiments show that DAAB has a stable structure. One reason may be that the surface plasmon decayed hot electrons have a higher energy to overcome the barrier, while the surface plasmon decayed holes cannot diffuse to the molecule (i.e., the electrons in the molecule transfer back). Therefore, reduction can occur much more easily than oxidation. The nitro groups of DNAB and NAAB can be reduced to amine NH_2_ groups by plasmon-driven catalysis.

**Figure 10:**
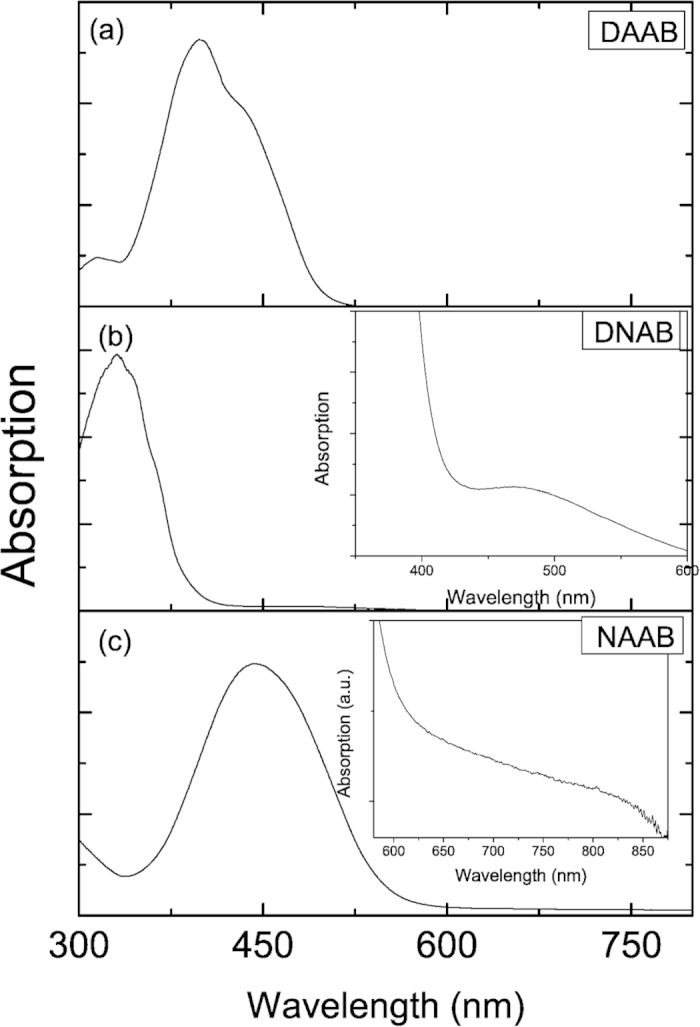
Absorption spectra of (**a**) DAAB, (**b**) DNAB, and (**c**) NAAB.

When the potential was 0 V, the plasmon-driven chemical reaction occurred for NAAB when excited by the 532 nm laser, while for DAAB, such a chemical reaction did not occur. The reason for this is that the molecular resonance may play an important role (Fig. 10). Figure 10(a) shows the absorption spectrum of DAAB, which reveals that the SERS excited at 532 nm is the normal Raman scattering. By contrast, Fig. 10(b) demonstrates that the SERS of DNAB excited at 532 nm is a pre-resonance Raman scattering, while the SERS of NAAB is a resonance Raman scattering excited at 532 nm (see Fig. 10(c)). Therefore, molecular resonance can also significantly enhance plasmon-driven chemical reactions. To further reveal the contributions of the molecular resonance absorptions to the plasmon-driven chemical reactions, we measured the electrochemical SERS spectra of NAAB (see Fig. 8) excited at 785 nm, where 785 nm is far from the SPR resonance of the roughened substrate (see Fig. 1d in Reference^17^); there was no chemical reaction at 0 V. With the increase of the external electric voltage, it is closer to the SPR peak^17^. Although the SPR is still weak at −0.6 V (see Fig. 6 in Ref. 17), the plasmon-driven chemical reaction for NAAB still occurred, reducing it to DAAB (see Fig. S2). This is direct evidence for molecular resonance in the plasmon-driven chemical reaction. At 785 nm, it is pre-resonant for NAAB, as shown in the inset in Fig. 10(c).

The plasmon-driven selective surface catalysis in our experiment is driven by three factors: the applied potential, the resonance of the surface plasmons and the resonance of the molecules. The applied potential will raise the Fermi level of the metal surface and will allow the electrons to overcome the meta-molecule junctions. The resonant plasmons will decay to hot electrons that can also overcome the junction barrier. The plasmon-decayed hot electrons should have higher energy (in our experiment, the measured range is approximately 1.3 ~ 2.7 eV) than the applied voltage (0 ~ –1.2 V). Thus, the surface plasmon plays the main role. Under some conditions, the applied voltage is not even necessary. However, they are associated with each other^16^. The resonance of the molecules overlapping with the surface plasmon resonance may increase the electron tunneling and transition because a Förster resonance energy transfer occurs, and the excited states of the molecules may be more easily reduced. The underlying mechanism still remains to be determined.

In summary, plasmon-driven selective surface catalytic reactions have been revealed by surface-enhanced Raman scattering in an electrochemical environment. Our experimental and theoretical evidence revealed the reduction of nitro groups to amine groups under plasmon conditions using electrochemical SERS spectra. However, the plasmon-driven oxidation of amine groups to nitro groups does not occur. The results reveal that the molecular resonance electronic absorption also plays an important role in the plasmon-driven chemical reaction. Our results are very interesting for plasmon chemistry and are of significant importance in elucidating the competition between reduction and oxidation in plasmon-driven chemical reactions in aqueous environments. Our results also provide us with a deeper understanding of plasmon-driven catalytic reactions. This method can be applied to other similar molecules and reactions.

## Methods

The Ag electrode (a single-crystal silver rod of 99.99% purity) was polished with emery paper and cleaned with Milli-Q water in an ultrasonic bath. Next, the electrode was placed in a typical electrochemical cell containing a solution of 0.1 M Na_2_SO_4_ for roughening. A double potential step was used to roughen the surface by applying a voltage of +0.25 V for 8 s and then applying a voltage of −0.35 V. This roughening treatment was performed to enhance the Raman intensity for the convenience of spectral recording^18^.

The DAAB and NAAB were purchased from Aldrich Chemical Co., Alfa Co. and Sigma Co. The DNAB was synthesized by Beijing Kaida Co. according to the customer’s requirements, and their NMR spectrum can be observed in Figures S3-S5 in the supporting information. The Raman spectra of the DNAB, DAAB and NAAB powders and their SERS spectra were recorded using a microprobe Raman system RH13325 (R-2000) spectrophotometer. For the SERS measurements, the applied voltage of the working electrode was controlled by a CHI619B electrochemical instrument. The samples were excited with 532 nm and 785 nm lasers with an effective power of 0.3 mW.

The theoretical calculations of the molecular Raman spectra and their vibrational modes were performed using Gaussian 09 software with density functional theory^19^, the pw1pw91 functional^20^, and the 6-31G(d) basis set. The pw1pw91 functional is the best functional for the calculation of the Raman spectra of azo compounds^21^.

## Additional Information

**How to cite this article**: Cui, L. *et al.* A plasmon-driven selective surface catalytic reaction revealed by surface-enhanced Raman scattering in an electrochemical environment. *Sci. Rep.* **5**, 11920; doi: 10.1038/srep11920 (2015).

## Supplementary Material

Supplementary Information
