# Supplementary material for: A plasmon-driven selective surface catalytic reaction revealed by surface-enhanced Raman scattering in an electrochemical environment
Source: Sci Rep. 2015 Jul 6;5:11920. doi: 10.1038/srep11920 (PMC4491720; doi:10.1038/srep11920)
Supplement: Supplementary Information [file srep11920-s1.pdf]

Supporting information

A plasmon-driven selective surface catalytic reaction revealed by surface-enhanced Raman scattering in an electrochemical environment

Lin Cui,[1,2] Peijie Wang,[1,*] Y. R. Fang,[3] Yuanzuo Li,[4] Mengtao Sun[2,*]

1. The Beijing Key Laboratory for Nano-Photonics and Nano-Structure, Center for Condensed Matter Physics, Department of Physics, Capital Normal University, Beijing 100048, People's Republic of China
2. Beijing National Laboratory for Condensed Matter Physics, Institute of Physics, Chinese Academy of Sciences, P. O. Box 603-146, Beijing, 100190, People's Republic of China
3. Division of Bionanophotonics, Department of Applied Physics, Chalmers University of Technology, Gothenburg SE-412 96, Sweden
4. College of Science, Northeast Forestry University, Harbin 150040, China

* Corresponding authors. Email: mtsun@iphy.ac.cn (M. Sun) or pjwang@cnu.edu.cn (P. Wang.).

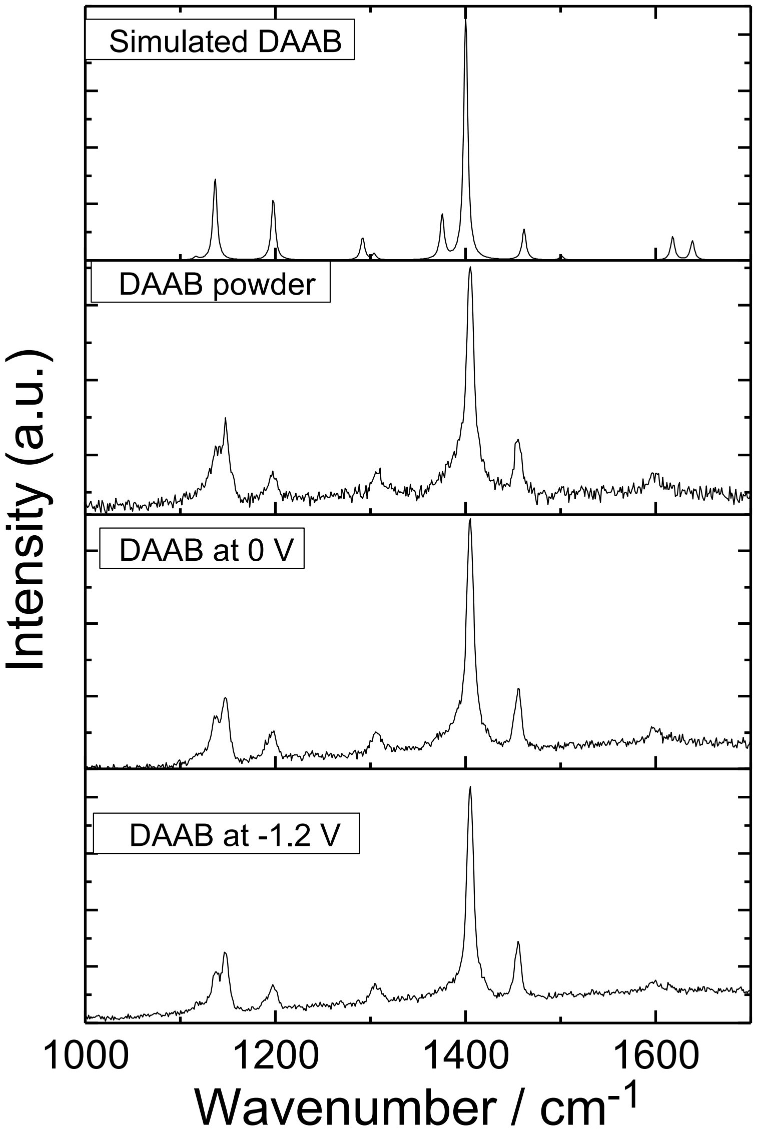

Figure S1 | Comparisons of Experimental and theoretical Raman spectra of DAAB.

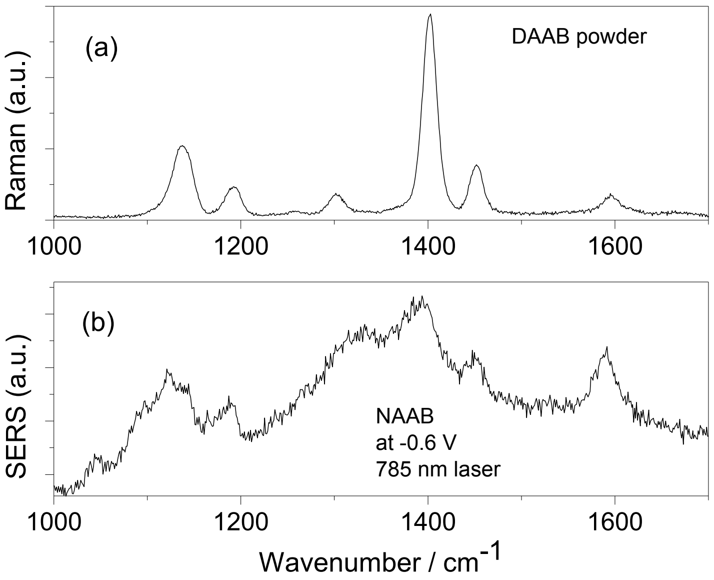

Figure S2 | Comparisons of Experimental Raman spectra. (a) Normal Raman spectrum of DAAB powder, and (b) the SERS of NAAB at -0.6 v, excited by 785 nm laser.

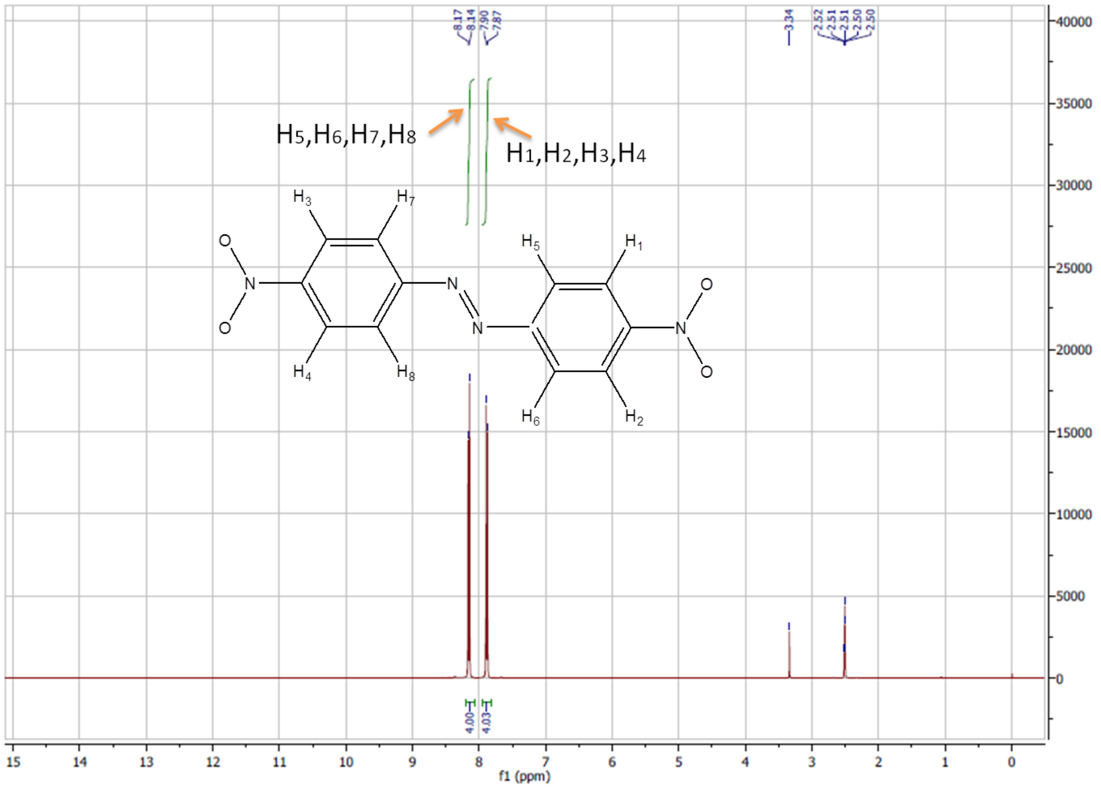

Figure S3 | NMR spectrum of DNAB.

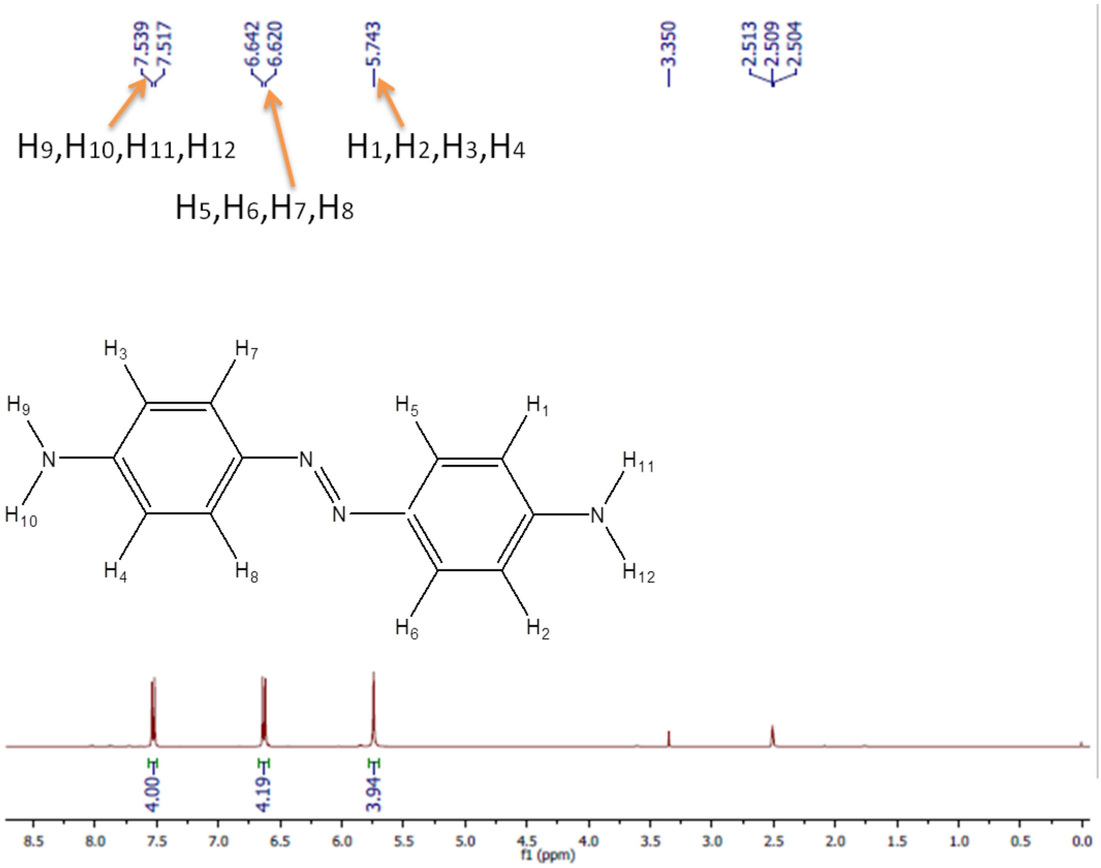

Figure S4 | NMR spectrum of DAAB.

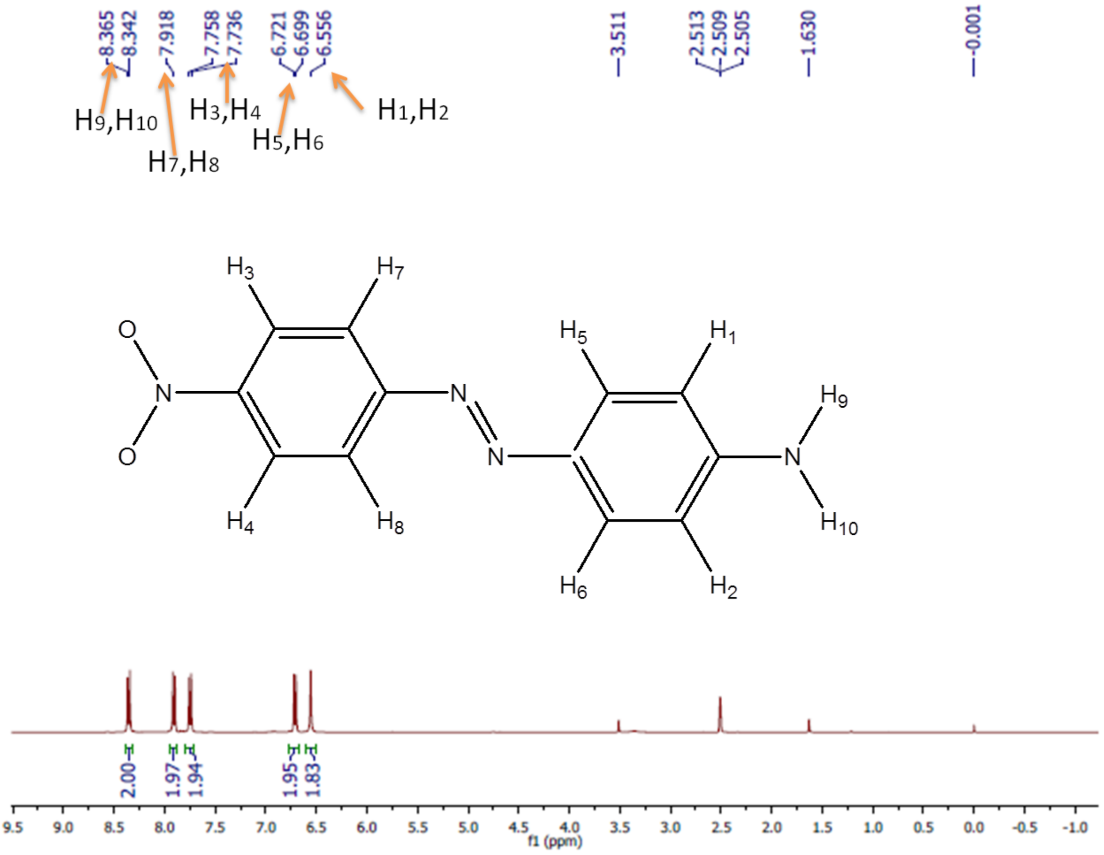

Figure S5 | NMR spectrum of NAAB.
